# A Dilemma in the Management of Distal Tibia Fractures Solved by Minimally Invasive Percutaneous Plate Osteosynthesis Technique: A Prospective Study

**DOI:** 10.7759/cureus.62777

**Published:** 2024-06-20

**Authors:** Jerin Jeevo, Rajagopal HP, Akhshay J George, Anoop Pilar, Madan Mohan Muniswamy, Binu Kurian, Mallikarjunaswamy Basappa, Rajkumar Amaravati, John Adarsh, Merwin Thomas

**Affiliations:** 1 Department of Orthopedics, St. John's Medical College Hospital, Bangalore, IND

**Keywords:** minimally invasive percutaneous plate osteosynthesis, locking compression plate, indirect reduction, distal tibia fractures, biological fixation, mippo

## Abstract

Introduction

Managing distal tibia fractures is challenging for trauma surgeons because of their peculiar anatomy with less soft tissue coverage and poor blood supply. There are various treatment options for distal tibia fractures such as open reduction and plating, minimally invasive percutaneous plate osteosynthesis, and intramedullary interlocking nailing. Open reduction and internal fixation can lead to excessive soft tissue dissection and devascularization of fracture fragments. We conducted a study on the functional outcome of distal tibia fractures treated by biological fixation with minimally invasive percutaneous plate osteosynthesis.

Methods

A total of 23 patients with distal one-third tibia fractures, fulfilling the inclusion criteria, who were treated at St. John's Medical College Hospital with minimally invasive percutaneous plate osteosynthesis between November 2020 and November 2022 were studied using the American Orthopaedic Foot & Ankle Society (AOFAS) score at six weeks, three months, and six months postoperative follow-up.

Results

This study included 17 males and six females. The mean age of the study participants was 43.78 years, with most of the participants being in the age group between 51 and 60 years (29.2%, n = 7). All the study participants were employed. The mean operative time was two hours and 10 minutes. The mean duration for the radiological union was 22 weeks. The mean AOFAS score at six months was 92.43 + 5.696. There was only one case of superficial infection, which was treated with intravenous antibiotics. There were no cases of malunion/nonunion.

Conclusion

Minimally invasive percutaneous plate osteosynthesis is an effective treatment for distal tibia fractures avoiding most of the complications such as wound dehiscence and malunion/nonunion involved in conventional open reduction and internal fixation with plating. Therefore, we recommend this technique for all distal tibia fractures.

## Introduction

In the lower extremity, 10% of fractures involve the tibial plafond. Axial forces with high energy or low energy can both produce fracture. They are frequently associated with severe closed soft tissue injury or open wounds [1]. The distal tibia is different from other body parts in such a way that it is located so near the ankle joint and has little soft tissue cover and scarce vascularity. The unanticipated wound complications that commonly accompany the surgical management of distal tibia fractures have been frustrating and trapping trauma physicians [2].

The classical open reduction and internal fixation (ORIF) with a plate, in particular, frequently involves considerable soft tissue dissection and substantial risks of sequelae, such as infection, skin necrosis, and delayed union or nonunion [3].

The preservation of the biological milieu at the fracture site was highlighted and is now thought to be essential to the healing of fractures with the development of the theories of biological osteosynthesis. In current clinical practice, minimally invasive percutaneous plate osteosynthesis (MIPPO) and intramedullary interlocking nailing (IMILN) are the favored treatments for extra-articular distal tibia fractures because they reduce damage to the wounded zone and maintain circulation around the fracture site. Numerous studies have compared the two treatment options for distal tibia fractures. However, there are conflicting reports about which fixation technique is superior [4]. Nonoperative care for this type of fracture frequently results in aggravating deformity and inescapable ankle stiffness, so it is not advised as the best course of action [5]. There are many techniques to treat distal tibia fractures, but the best mode of treatment is still debatable [6].

## Materials and methods

A total of 23 patients with distal one-third tibia fractures, fulfilling the inclusion criteria, who were treated at St. John's Medical College Hospital with MIPPO between November 2020 and November 2022 were studied.

Inclusion criteria included patients between the age group of 18 and 90 years, and patients with the following fracture morphology: extra-articular distal tibia fractures (Arbeitsgemeinschaft für Osteosynthesefragen/Orthopedic Trauma Association (AO/OTA) A1, A2, A3), partial articular fractures (AO/OTA B1, B2, B3), and intra-articular fractures (AO/OTA C1 C2).

Exclusion criteria included AO type C3 fracture, compound fractures/open fractures, old fractures, and fractures with neurovascular injuries or compartment syndrome.

When the patients came to the emergency department/OPD, a primary and secondary survey was done, followed by stabilization of the lower limb with a below-knee slab. After that, we took anteroposterior (AP) and lateral radiographs of the patient's ankle and leg. On admission to the ward, a complete medical history was collected, following which a detailed clinical examination was done. For the study, institutional ethics committee approval was obtained. Before the surgical procedure and for the research participation, informed consent was acquired from each patient.

Standard operative technique was used for all our cases and fracture reduction was done under the guidance of C-arm. Less than 5 degrees of varus-valgus angulation, less than 10 degrees of anterior-posterior angulation, and less than 15 mm of shortening were considered acceptable reductions. Once the reduction was satisfactory, the fracture was stabilized with the locking plate under the guidance of the C-arm.

Operative technique

The patient was positioned supine on the operation table under spinal or general anesthesia, with a sandbag beneath the injured side gluteal region. Time was recorded after applying a pneumatic tourniquet to the proximal thigh. The affected limb was painted with povidone-iodine solution and draped. Surgery duration was about 1-1 ½ hours and MIPPO was performed. The decision on whether to fix the fibula was made by the operating surgeon to achieve proper alignment of the leg.

Approach to Distal Tibia

**Figure 1:**
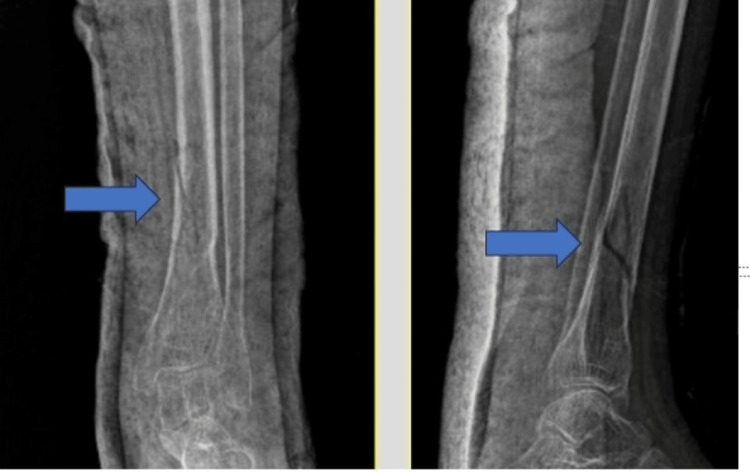
Radiograph showing extra-articular distal tibia fracture. Pointer highlighting extra-articular distal tibia fracture in anteroposterior and lateral radiographs.

A patient with extra-articular distal tibia fracture is explained here as an example (Figure 1). This patient had a history of a road traffic accident and sustained a closed injury to the leg. The patient was taken up for surgery once the swelling subsided (three days).

**Figure 2:**
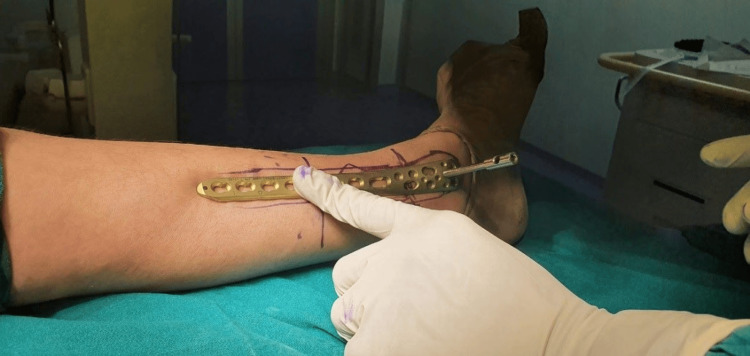
Marking skin incision based on the plate placement (intraoperative).

Taking into account where the plate was situated in vitro, two 3-4 cm longitudinal incisions were made in the skin beneath the two ends (Figure 2).

**Figure 3:**
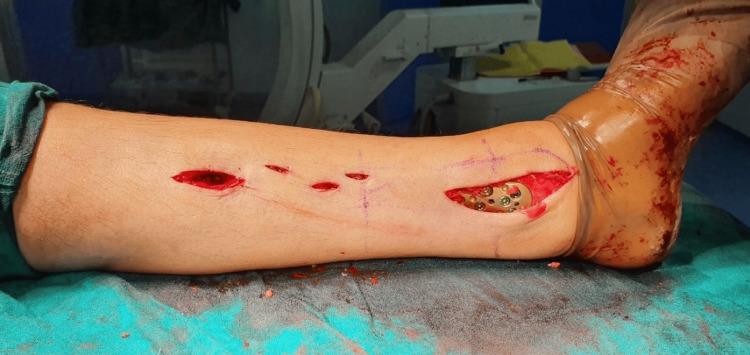
Intraoperative image showing the plate placement through the distal incision.

Two incisions were made, one at the proximal end of the plate along the medial portion of the tibia and the other at the midline of the medial malleolus. Then, employing blunt dissection, a subcutaneous extra-periosteal tunnel could be created between these two incisions. The distal tibia locking plate was inserted percutaneously from distal to proximal while the great saphenous vein was protected (Figure 3).

**Figure 4:**
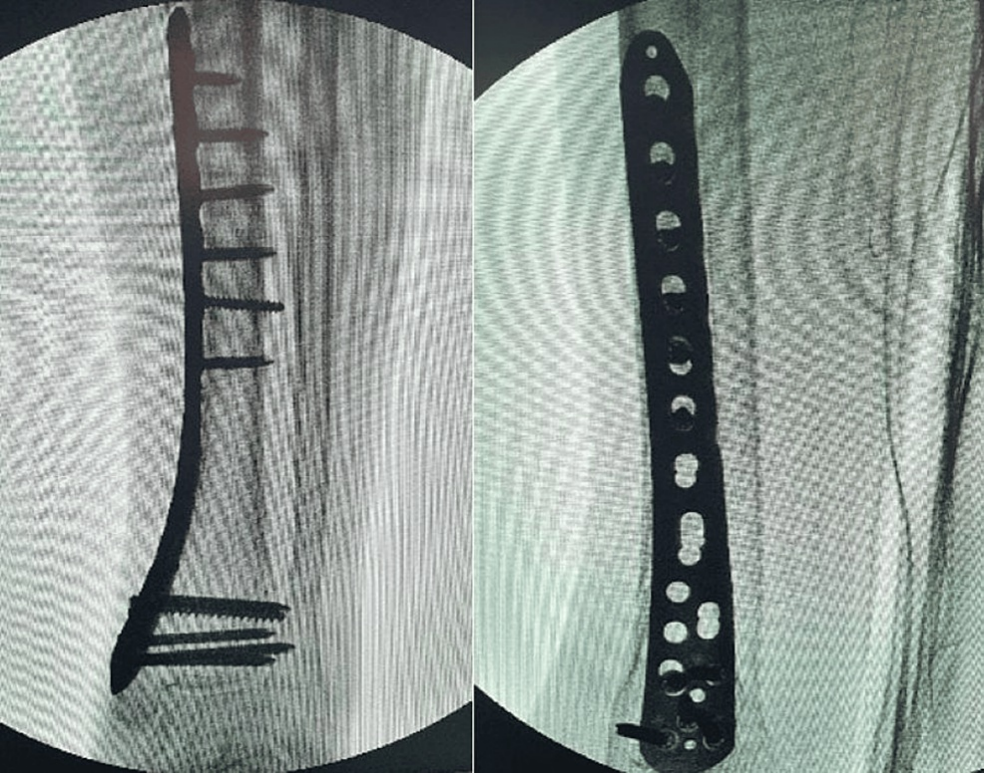
Intraoperative C-arm image showing distal tibia locking plate and screws at the completion of the procedure.

The assistant used traction (indirect reduction) to correct the length and coronal alignment of the fractured leg with the help of fluoroscopy. Once the fracture was reduced, the plate location was modified, and locking screws were used to secure the bone and plate (Figure 4).

**Figure 5:**
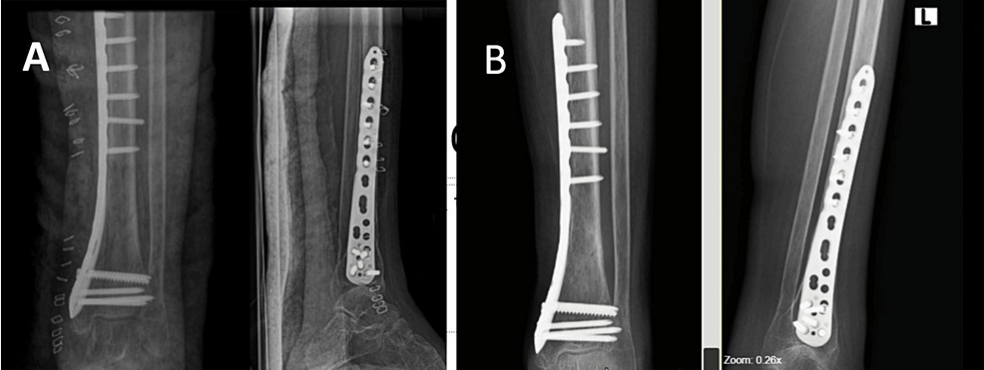
Postoperative radiographs. (A) Postoperative day one. (B) Six months post operation.

The postoperative radiograph shows rigid fixation with good alignment of the distal tibia. The radiograph taken at six months follow-up shows the complete union of the fracture (Figure 5).

Postoperative management

Intravenous fluids were given during the nil per oral period. Intravenous antibiotics containing cefoperazone and sulbactam were given for five days. Analgesics were given. The operated limb was elevated with pillows. Postoperative radiographs (AP and lateral) were taken. A wound inspection was done on the second postoperative day. Sutures were removed on the 12th postoperative day on average. Below knee slab was applied for all cases to protect the fixation and patients were discharged with instruction of non-weight bearing crutch walk for a period of six weeks and to come for follow-up. Ankle range of motion was started at three weeks post operation. Patients were followed up at six weeks, three months, and six months and were evaluated using the American Orthopaedic Foot & Ankle Society (AOFAS) score introduced by Kitaoka et al. [7]. In this scale, both subjective and objective variables were used and they were classified into three main categories, which consisted of pain (40 points), function (50 points), and alignment (10 points). Based on the total AOFAS score at each follow-up visit, four outcomes (excellent, good, acceptable, and bad) were derived (Table 1) [8].

**Table 1:** AOFAS score and outcome. AOFAS: American Orthopaedic Foot & Ankle Society.

AOFAS score	Outcome
>89	Excellent
80-89	Good
70-79	Acceptable
<69	Bad

## Results

This study involved a total of 23 participants, which included 17 males and six females. In this study, the mean age of the participants was found to be 43.78 years. Most of the patients were in the age group between 51 and 60 years (29.2%, n = 7). All the participants involved in this study were employed. The most common mode of injury was road traffic accidents (78.26%, n = 18). Most of the study participants had extra-articular fractures - AO/OTA 43 type A (78.2%, n = 18) (Table 2).

**Table 2:** Classification of fractures. AO/OTA: Arbeitsgemeinschaft für Osteosynthesefragen/Orthopedic Trauma Association.

Fracture classification – AO/OTA 43	Percentage
Extra-articular – Type A	78.2 % (n = 18)
Partial articular – Type B	8.6 % (n = 2)
Complete articular – Type C	13.04 % (n = 3)

The mean duration between trauma and surgery was 3.52 days with a range of two to seven days. In our study, the mean operating time was two hours and 10 minutes and the mean duration to achieve radiological union was 22 weeks. Among the study participants, 10 were smokers and the mean duration to achieve union among them was 26 weeks, which is more than the non-smokers (19 weeks). The mean AOFAS total scores at three and six months were 74.46 + 8.278 and 92.43 + 5.696, respectively (Tables 3, 4).

**Table 3:** Follow-up findings at three months – AOFAS score. AOFAS: American Orthopaedic Foot & Ankle Society.

Domains	Minimum	Maximum	Mean	SD
Pain	20	40	29.13	5.964
Activity limitations	4	10	6.48	1.729
Maximum walking distance	2	4	3.83	0.576
Walking surface	3	5	3.26	0.689
Gait	4	8	5.22	1.882
Sagittal motion	4	8	5.22	1.882
Hindfoot motion	0	6	3.26	1.544
Ankle hindfoot instability	8	8	8.00	0.000
Alignment	10	10	10.00	0.000
Total score	55	86	74.46	8.278

**Table 4:** Follow-up findings at six months – AOFAS score. AOFAS: American Orthopaedic Foot & Ankle Society.

Domain	Minimum	Maximum	Mean	SD
Pain	30	40	38.70	3.444
Activity limitations	4	10	8.43	1.779
Maximum walking distance	4	5	4.70	0.470
Walking surface	3	5	4.22	0.998
Gait	4	8	6.78	1.882
Sagittal motion	4	8	6.78	1.882
Hindfoot motion	0	6	4.83	1.749
Ankle hindfoot instability	8	8	8.00	0.000
Alignment	10	10	10.00	0.000
Total score	76	100	92.43	5.696

There was no significant association found between total AOFAS score and type of fracture at six weeks and six months but a significant association was found at three months (Table 5).

**Table 5:** Classification of fractures compared with total scores during follow-ups. A1: simple extra-articular distal tibial fracture; A2: distal tibial extra-articular wedge fracture; A3: multi-fragmentary extra-articular fracture of the distal tibia; B1: pure split fracture; C1: simple articular and metaphyseal fracture; C2: simple articular and multi-fragmentary metaphyseal fracture.

Classification		6 weeks	3 months	6 months
A1	Mean	53.89	77.56	92.56
	SD	9.307	6.912	4.216
A2	Mean	48.60	66.40	90.00
	SD	17.199	8.173	8.860
A3	Mean	53.00	78.00	96.00
	SD	1.155	5.774	1.155
B1	Mean	60.00	82.50	98.50
	SD	7.071	2.121	2.121
C1	Mean	51.00	75.00	91.00
	SD	.000	1.414	2.828
C2	Mean	42.50	65.00	86.50
	SD	3.536	7.071	3.536
P-value		0.597	0.023	0.186

**Figure 6:**
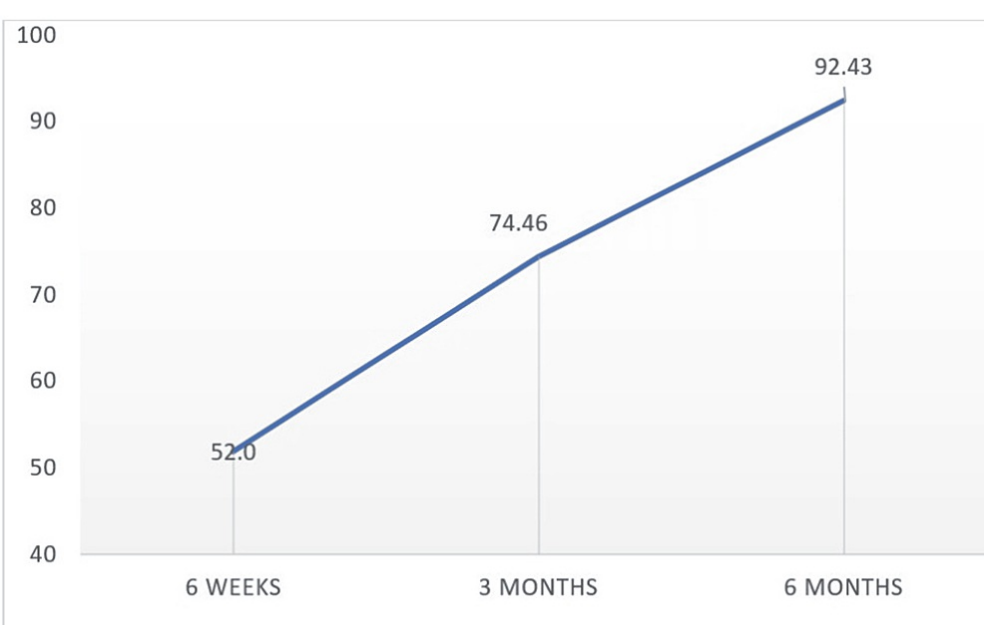
Functional outcomes during the follow-up.

In our study, 69.56% of the study participants who had bad functional outcomes at six weeks had excellent functional outcomes at six months, followed by 26.08% having good functional outcomes. The mean functional outcome was found to increase with each follow-up visit (Figure 6).

In our study, only one patient developed a superficial infection, which was treated with daily dressing and IV antibiotics. The wound was healthy after two weeks of treatment. None of the study participants developed malunion/nonunion (Table 6).

**Table 6:** Complications.

Complications	Percentage
Superficial infection	4.3 % (n = 1)
Malunion/nonunion	0

## Discussion

In ORIF, there is extensive soft tissue stripping and drainage of fracture hematoma to achieve anatomical reduction. There is also an increased risk of infection, delayed union, and nonunion. There should be a balance between soft tissue stripping and anatomical reduction to minimize these complications. There is a shift from the traditional concept of absolute stability to the biological concept of indirect reduction and relative stability. In minimally invasive surgeries, instead of opening the fracture site, indirect reduction techniques with the help of fluoroscopy are utilized [9].

Intramedullary interlocking nailing for distal tibia fractures results in reduced mechanical stability because of the wide distal metaphyseal area of the tibia in relation to the nail diameter [10]. Recently developed tibial nail constructs provide interlocking holes close to the tip of the nail but these screws do not provide adequate purchase to the metaphyseal bone. Intramedullary nails are associated with high rates of implant failure at the distal locking sites due to loss of reduction as a late complication [11]. There are also reports of anterior knee pain and fracture malalignment with the use of intramedullary interlocking nailing [12]. The MIPPO technique provides a favorable biological environment for fracture healing by reducing the soft tissue dissection thereby preserving the blood supply [13].

The choice of fixation is also influenced by biomechanical considerations, particularly when stabilizing unstable fractures with plates. To protect the fracture and withstand outside stresses while osseous consolidation takes place, optimal plate fixation should incorporate the strength and stability provided by the implant [12]. For distal tibia fractures, Yenna and colleagues assessed the biomechanical characteristics of medial and anterolateral plates. Saw bone models, designed to simulate AO/OTA type 43 A2 fractures, were used to evaluate plate stiffness under both axial and torsional loading. They discovered that when external compression or torsional forces were applied, there was no apparent difference in stiffness between the medial and anterolateral plate designs [14].

Positive outcomes for MIPPO for distal tibia fractures have been documented in the literature. The use of MIPPO for distal tibia fractures has been associated with outcomes that include high union rates and fewer cases of malalignment [15-17].

In this study, the mean AOFAS total score at three months was found to be 74.46 + 8.278, which was almost similar to a study by Illur et al. [8] (Table 7).

**Table 7:** Total AOFAS score at three months compared with other studies. AOFAS: American Orthopaedic Foot & Ankle Society.

Study	Total score at 3 months
Illur et al. [8]	74.39
Collinge et al. [16]	85
Guo et al. [18]	83.9
This study	74.46

The mean AOFAS total score at six months was found to be 92.43 + 5.696. Paluvadi et al. [10] reported a mean AOFAS score of 95.06, which was similar to this study. Ahmad et al. [15] and Kariya et al. [17] reported a mean total AOFAS score of 88.8 and 79.8, respectively, which is less compared to this study (Table 8).

**Table 8:** Total AOFAS score at six months compared with other studies. AOFAS: American Orthopaedic Foot & Ankle Society.

Study	Total score at 6 months
Paluvadi et al. [10]	95.06
Ahmad et al. [15]	88.8
Guo et al. [18]	83.9
Kariya et al. [17]	79.8
This study	92.43

The mean duration for radiological union in this study was 22 weeks, which is similar to other studies (Table 9). Fracture is said to be united when the patient is able to walk while bearing full weight on the affected leg without pain and radiological consolidation of three out of four cortices in anteroposterior and lateral radiographs. The mean duration for radiological union in smokers (26 weeks) is more than in non-smokers (19 weeks), which is statistically significant. All the participants in this study were able to return to their pre-injury occupations and other daily activities.

**Table 9:** Mean duration of union in this study compared to other studies.

Study	Mean time to union (in weeks)
Illur et al. [8]	22
Paluvadi et al. [10]	21.6
Collinge et al. [16]	21
Guo et al. [18]	17.6
This study	22

The limitations of our study are that the number of participants was low and there was no control group for comparison.

## Conclusions

The indirect reduction technique used in MIPPO leads to reduced soft tissue stripping and preservation of blood supply, which hastens fracture union. We also found a significant association between smokers and delayed fracture union. The mean functional outcome was found to increase with each follow-up visit and there were no serious complications. The main focus of the study is the absence of deep infection, high union rates, and early mobilization of the ankle with the MIPPO technique. Therefore, we recommend the MIPPO technique for distal tibial fractures to get a favorable postoperative outcome.
